# A new mixture model approach to analyzing allelic-loss data using Bayes factors

**DOI:** 10.1186/1471-2105-5-182

**Published:** 2004-11-24

**Authors:** Manisha Desai, Mary J Emond

**Affiliations:** 1 Department of Biostatistics, Columbia University, 722 West 168th Street, R629, New York, NY 10032, USA; 2 Department of Biostatistics, University of Washington, Box 357232, Seattle, Washington 98195, USA

## Abstract

**Background:**

Allelic-loss studies record data on the loss of genetic material in tumor tissue relative to normal tissue at various loci along the genome. As the deletion of a tumor suppressor gene can lead to tumor development, one objective of these studies is to determine which, if any, chromosome arms harbor tumor suppressor genes.

**Results:**

We propose a large class of mixture models for describing the data, and we suggest using Bayes factors to select a reasonable model from the class in order to classify the chromosome arms. Bayes factors are especially useful in the case of testing that the number of components in a mixture model is *n*_0 _versus *n*_1_. In these cases, frequentist test statistics based on the likelihood ratio statistic have unknown distributions and are therefore not applicable. Our simulation study shows that Bayes factors favor the right model most of the time when tumor suppressor genes are present. When no tumor suppressor genes are present and background allelic-loss varies, the Bayes factors are often inconclusive, although this results in a markedly reduced false-positive rate compared to that of standard frequentist approaches. Application of our methods to three data sets of esophageal adenocarcinomas yields interesting differences from those results previously published.

**Conclusions:**

Our results indicate that Bayes factors are useful for analyzing allelic-loss data.

## Background

### Allelic-loss data

The goal of studies of allelic loss is to determine those loci in tumor tissue where genetic material has been lost. A tumor suppressor gene (TSG) is much more likely to lie on a chromosome arm where there has been significant allelic loss than elsewhere [1,2]. The statistical challenge lies in distinguishing between "random" allelic loss that is expected in a tumor cell population and "nonrandom" loss that may be biologically meaningful. This corresponds to determining whether there is one group of arms with background allelic loss versus two groups of arms, one with background loss rates and one with elevated loss rates.

### Three allelic-loss data sets on esophageal adenocarcinomas

Esophageal adenocarcinoma is a form of cancer involving the cells along the lining of the esophagus. The cause of esophageal adenocarcinoma is not well understood. The incidence of this cancer has been increasing rapidly. In fact, it is one of the fastest growing cancers in the United States over the past 20 years [1,3,4]. A strong association has been established between the pre-malignant condition known as Barretts esophagus and the development of adenocarcinomas of the esophagus. Barretts esophagus is a condition that develops in 10–20% of patients with chronic gastroesophageal reflux disease. The condition is characterized by the metaplastic change from normal squamous to columnar epithelium in the esophagus [1,4]. Approximately 1% of patients with Barretts esophagus progress to esophageal cancer [3]. Of those who develop the cancer about 90% will die as a result of the disease [1].

We examine three data sets of allelic-loss on esophageal adenocarcinomas that attempt to identify the tumor suppressor genes (TSGs) involved in the development of this disease. These data sets have been previously analyzed and published. We refer to each data set by the last name of the first author of the publication. Some of the data sets record allelic loss on multiple loci per chromosome arm for some of the arms. However, because the number of loci evaluated per chromosome arm is not random (i.e., chromosome arms suspected of harboring a TSG will be assessed at more loci than others), we consider only one locus per chromosome arm. In these cases, we choose data from the most informative locus for that chromosome arm.

### Our approach

Our general approach to analyzing allelic-loss data can be described in two main steps. The first step is to choose an appropriate model for the data using Bayes factors. The second step is to classify the chromosome arms as harboring TSGs or not according to the selected model. The details involved in these two steps are described below.

## Results and Discussion

### Proposed class of models

A natural way to model allelic-loss data is in terms of a mixture of two distributions: one distribution corresponds to chromosome arms that harbor TSGs and the other corresponds to arms that do not. It is reasonable to expect considerable variability in the loss rates of arms that harbor TSGs due to the existence of multiple pathways leading to the same tumor type [5]. For example, deletion of a particular TSG may be in the causal pathway for 60% of tumors of a particular type while another TSG (or other TSGs) may account for the remaining 40% of the cases. In addition, it is conceivable that various factors play a role in background loss rates. For example, factors such as cell viability, fragility of the chromosome arm, and the length of telomeres are believed to influence background loss rates [6]. It is plausible that the non-TSG loci that contribute to the background loss rate are in fact composed of two biologically different groups of loci. This group includes loci that are essential for cell viability and those that are not essential. The essential loci would be expected to exhibit loss rates considerably lower than that of the non-essential loci as their function controls the cell's survival.

We propose a class of mixture models that account for the variation inherent in this type of data. Specifically, the class of models we propose is a mixture of two beta-binomial distributions. Let *X*_*i *_be the number of tumors with allelic-loss for the *i*th chromosome arm, and let *n*_*i *_be the number of informative tumors for the *i*th chromosome arm, for *i *= 1, 2,...,*N*, where *N *is the number of chromosome arms in the study. The density function for *X*_*i *_is written as follows:


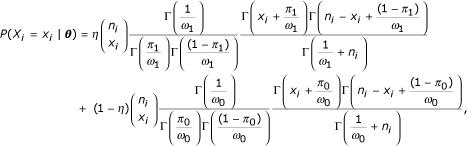


where ***θ ***≡ (*η*, *π*_1_, *ω*_1_, *π*_0_, *ω*_0_) is a vector of unknown parameters, *η *is the mixing probability, *π*_*j *_is the average loss rate, and *ω*_*j *_is the dispersion parameter for *j *= 0,1.

The distribution converges to a mixture of two binomial distributions as both dispersion parameters go to 0 (*ω*_0 _→ 0 and *ω*_1 _→ 0). If only one of the dispersion parameters goes to 0 (*ω*_0 _→ 0 or *ω*_1 _→ 0), the distribution reduces to a mixture of a beta-binomial and a binomial distribution. Note that the model has only one component when the mixing parameter is zero (*η *= 0).

### Model selection using Bayes factors

Bayes factors are measures used to compare the fit of two competing models. We suggest using Bayes factors to select an appropriate model for the data from the proposed class of mixture models. Let H_0 _and H_1 _represent the models under the null and alternative hypotheses, respectively. When comparing two models, it is of interest to examine the posterior odds of one model to another. It is easy to show that the posterior odds of one model to another is


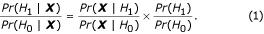


Equation (1) shows that the posterior odds is calculated as the product of a term known as the Bayes factor and the prior odds. The Bayes factor is the marginal likelihood of the data under *H*_1 _divided by the marginal likelihood of the data under *H*_0_, or *B*_10 _≡ *Pr*(***X ***|*H*_1_)/*Pr*(***X***|*H*_0_). Thus, as Bayes factors are proportional to the posterior odds of one model to another, they are desirable measures to use for model selection. Note that if the prior odds are assumed to be 1, then the Bayes factor is equivalent to the posterior odds.

One can think of the Bayes factor as a Bayesian likelihood ratio statistic. Like the likelihood ratio statistic, the Bayes factor is a ratio of likelihoods under two models being considered. However, while the likelihood ratio statistic is the ratio of two maximized likelihoods for two competing, nested models, the Bayes factor is the ratio of two likelihoods integrated or averaged over the entire parameter space and the models need not be nested. An important consideration with a Bayesian approach is that a prior distribution is assumed for all of the parameters in the model. The advantage to this is that one can incorporate prior information into determining which model is more appropriate. This is a disadvantage, however, if the Bayes factor is sensitive to the prior and if the prior has been chosen incorrectly.

Large Bayes factors are evidence in favor of the alternative hypothesis. Kass and Raftery (1995) discuss guidelines for interpreting the measure [7]. Following the authors' suggestion, we transform the Bayes factor to the same scale as that of the likelihood ratio statistic and use the criterion that 2*lnB*_10 _> 2 implies positive evidence in favor of the alternative model.

Comparing a uni-component model to a two-component model would address the question of whether there is one versus two groups of chromosome arms. Further, comparing a two-component beta-binomial model to a two-component binomial model would address whether there is overdispersion in either group. The advantage of this is that it provides insight into the number of chromosome arm groups, whereas standard applicable frequentist tests will only indicate whether there is one or more groups [8,9].

### Classification

Provided there is sufficient evidence to indicate that there are two groups of chromosome arms, it is desirable to identify which chromosome arms belong in which group. Classification of the chromosome arms can be done by calculating the conditional probability of group membership of each arm under a given model. If *X*_*i *_~ *ηf*_1_(*x*_*i*_, *n*_*i*_, ***θ***_1_) + (1 - *η*)*f*_0_(*x*_*i*_, *n*_*i*_, ***θ***_0_), then it can be shown using Bayes' rule that


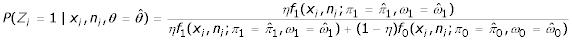


where 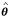 is the maximum likelihood estimate (MLE) of ***θ***, *Z*_*i *_is the group membership of the *i*th chromosome arm and *Z*_*i *_= 1 implies that the *i*th chromosome arm is in the TSG group. For the analyses here, chromosome arms with conditional probabilities exceeding 0.5 are classified in the TSG group. Also note that MLEs are computed using the nlminb function in S-Plus.

### Performance of the Bayes factors

Table 1 presents a description of simulated data sets used to evaluate the performance of the Bayes factors. One hundred data sets are generated under each scenario. All parameters chosen to generate the data are based on the Barrett esophageal cancer data set discussed later [1]. Under the first scenario, data are generated from a two-component binomial mixture model, where each group has a constant loss rate. The two groups are fairly well-separated with the TSG group's loss rate considerably higher than the background loss rate. We specify only five chromosome arms to harbor TSGs, which is believed to be typical. The second scenario is one where there are no TSGs and the background loss rate follows a beta distribution. The distributional parameters are chosen by examining the Barrett data set after removal of the five chromosome arms with the highest rates of allelic loss (these arms are implicated by Barrett et al. (1996) [1] as potentially harboring TSGs). This gives an expected loss rate of 0.26 and a dispersion parameter of 0.07. Under the third scenario there are two groups of chromosome arms with one group exhibiting a constant background loss rate of 0.22 and the second group of five chromosome arms exhibiting varying and higher rates of allelic loss. In the last scenario, both groups of chromosome arms have varying loss rates. The TSG loss rate distribution follows that of Scenario 3 and the non-TSG loss rate distribution follows that of Scenario 2.

Table 2 presents the percentage of time one model is favored over the other based on 2*ln*(Bayes factor) for data generated under each of the scenarios described in Table 1. For each scenario, a 5 × 5 matrix of pairwise comparisons is presented. The rows of the matrix correspond to models considered under *H*_1 _(models appearing in the numerator of the Bayes factor). The columns of the matrix correspond to models considered under *H*_0 _(models appearing in the denominator of the Bayes factor).

For data generated from a two-component binomial model (Scenario 1), the true model is mostly favored over the uni-component models. In fact, when comparing the true model to a uni-component beta-binomial model, the latter model is only favored 5% of the time. This can be viewed as a false-negative rate. Note that the Bayes factors never provide evidence in favor of a uni-component model in comparisons with either of the other two-component models for data from this scenario. Furthermore, the true model is selected 75% of the time over the two-component beta-binomial model. The Bayes factors are ambiguous, however, when comparing the true model to a two-component beta-binomial/binomial model, where neither is favored 69% of the time.

For data that follow a uni-component beta-binomial distribution (Scenario 2), the results are inconclusive 62% of the time when comparing the true model to the two-component binomial model. For twenty-two percent of the data sets the right model is favored, but 16% of the time, the two-component model is selected. Thus, this comparison results in a 16% false-positive rate. Similar results are found when comparing the true model to a two-component beta-binomial/binomial model. The Bayes factors favor the correct model over the two-component beta-binomial model roughly half the time and favor neither model the other half. Comparisons between the two-component models and the one-component binomial model not surprisingly show a strong preference for the two-component models, as they better accommodate the variability of the data.

The third quarter of Table 2 presents results for data generated under Scenario 3. The two-component beta-binomial/binomial model is favored in the majority of the cases over the other models within the class, which makes sense as this model is most similar to the data-generated model. Only once is an alternative hypothesis favored when compared to this model and this is the two-component beta-binomial model. When comparing the two-component beta-binomial/binomial model to the other two-component models, the Bayes factors do not favor either of the models being compared about 20 percent of the time. In general, the two-component models were mostly favored over the one-component models.

For data generated under Scenario 4, we expect the two-component beta-binomial model to be chosen over the other models in the class as this model is closest to the truth. The results show that when this model is compared to the two-component binomial or the one-component beta-binomial, it is mostly favored, and these models are never selected. As the two-component beta-binomial model is fairly similar to the two-component beta-binomial/binomial model, however, most of the time neither model is chosen over the other. The two-component beta-binomial is favored only 35% of the time, while the two-component beta-binomial/binomial is favored 9% of the time. Interestingly, when comparing the one-component beta-binomial to the two-component binomial, the one-component model is chosen 72% of the time and the two-component binomial model is chosen only 5% of the time. This suggests that the measure is fairly sensitive to the overdispersion in the two groups. Another example of this is a comparison between the two-component beta-binomial/binomial model and the one-component beta-binomial model. In this case, the two-component model is only favored 54% of the time, where the uni-component model is a better fit to 5% of the data sets, and both models are equally good fits to the data 41% of the time.

This simulation study demonstrates that the Bayes factors are an appropriate method of model selection. They perform particularly well for data generated from the two-component models. In particular, most of the time, the correct model is chosen, and furthermore, reasonable false-negative rates are observed for comparisons made on data generated from the two-component binomial model as well as the two-component beta-binomial/binomial model. Data generated from a one-component beta-binomial model produces interesting results. Although the false-positive rates are reasonable when comparing the one-component beta-binomial model to the other two-component models (16%, 7% and 0% for the two-component binomial, two-component beta-binomial/binomial, and two-component beta-binomial, respectively), there is a large percentage of time, when neither model is favored (62%, 69% and 50%). Since both models are often good fits to the data, it would be difficult to decide with confidence whether or not there is a second group of arms in these cases.

### Application of methods to data sets

In this section, we apply the methods discussed to three allelic-loss data sets. Specifically, we use Bayes factors to choose a reasonable model or set of models for the data in order to address whether TSGs exist on any of the chromosome arms, and we classify the chromosome arms as harboring TSGs or not based on the selected model(s).

Table 3 presents a summary of the results for the three data sets. The set of models chosen by the Bayes factors is provided along with the individual chromosome arms that were identified as having TSGs based on these models. The set of chosen models was comprised of those with 2*ln*(Bayes factors) exceeding 2 when compared to models outside the set and with 2*ln*(Bayes factors) less than 2 when compared to models within the set. Details of the analysis for each data set are described below, with slightly more emphasis placed on the first data set.

#### The Barrett data set

**Figure 1:**
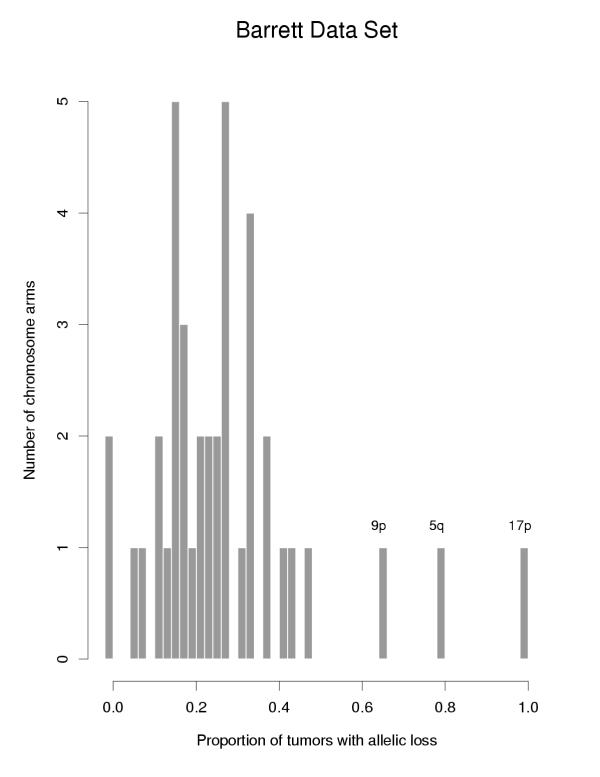
Histogram of allelic loss for the Barrett data set

The Barrett data set records allelic loss on 20 esophageal adenocarcinomas and two high-grade dysplasias. Figure 1 presents a histogram of the proportion of tumors with allelic loss for each of the forty chromosome arms studied (markers were not placed on the short arms of chromosomes 13, 14, 15 and 22 as these are too small to study). Two of the chromosome arms examined do not exhibit allelic loss (arms 20q and 21p) for any of the tumors observed. The mean allelic-loss rate for all arms exhibiting loss is 0.27 and the median allelic-loss rate is 0.24. From the figure, three chromosome arms appear to stand apart from the others in exhibiting considerably higher allelic-loss rates: 9p, 5q, and 17p.

Table 4 presents 2*ln*(Bayes factors) for the pairwise comparisons of the models for each of the three data sets. In addition, the posterior probability of each model is presented assuming a prior probability for the models such that

*P*(2 Component Model) = *P*(1 Component Model) = 1/2

This gives

*P*(2 bb) = *P*(2 bb/bin) = *P*(2 bin) = 1/6

*P*(1 bb) = *P*(1 bin) = 1/4.

For the Barrett data set, the two-component models are strongly favored over the one-component models, clearly indicating a group of arms that exhibit higher than background loss rates. In particular, the Bayes factors demonstrate that the two-component beta-binomial/binomial model provides the best fit. Note that the posterior probability of this model is considerably higher than that of the others, providing further evidence of its superiority.

Table 5 presents the MLEs of the parameters for the two-component models listed in order of posterior probability (largest to smallest). First note that 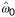 = 0, reducing the two-component beta-binomial model to a two-component beta-binomial/binomial model. The parameter estimates for these two models are identical and imply that the beta-binomial distribution corresponds to the TSG loss and the binomial distribution corresponds to the background loss. The estimate of the probability that a chromosome arm is in the TSG group is 0.097. The estimated background loss rate is 0.228, and the expected background loss rate for arms with TSGs is estimated at 0.708 with a loss rate variance of 0.07. The fit from the two-component binomial model gives a slightly lower mixing parameter estimate and a slightly higher estimate of the TSG loss rate.

The conditional probabilities of group membership based on the two-component beta-binomial/binomial model yield the same classification rule as that based on the other two-component models. Chromosome arms 5q, 9p, and 17p are classified in the TSG group. The conditional probabilities of group membership for these chromosome arms are quite similar across the three models.

#### The Gleeson data set

**Figure 2:**
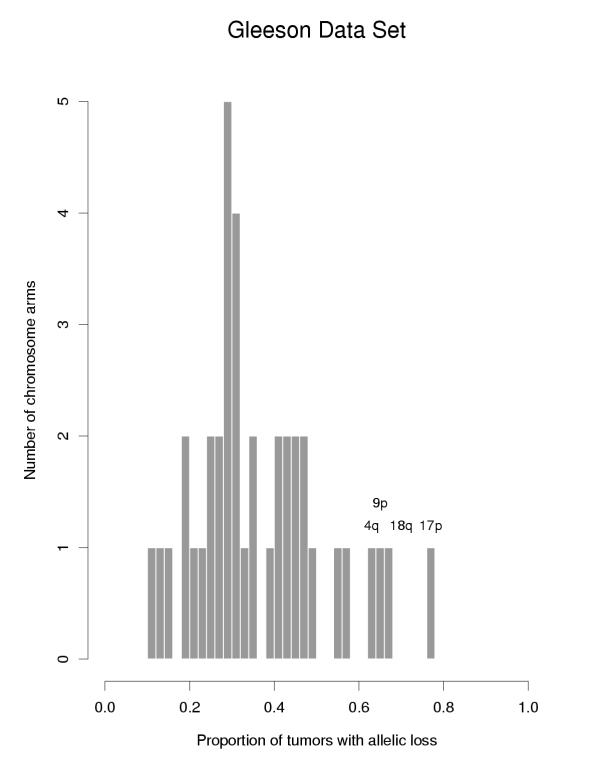
Histogram of allelic loss for the Gleeson data set

The Gleeson data set consists of 38 esophageal adenocarcinomas. Allelic-loss data were recorded on 39 chromosome arms (as in the Barrett data set, the short arms of chromosomes 13, 14, 15, 21, and 22 were not included in the study). A histogram of the proportion of tumors with allelic loss is presented in Figure 2. The mean allelic-loss rate is 0.36 and the median allelic-loss rate is 0.32. By simply viewing the histogram, four of the chromosome arms have been identified as having suspiciously high allelic-loss rates. These are chromosome arms 4q, 9p, 18q, and 17p.

For the Gleeson data set, the two-component beta-binomial/binomial model, the two-component binomial model and the uni-component beta-binomial model are all favored over the two-component beta-binomial model and the uni-component binomial model (See Table 4). Because two of the two-component models as well as the uni-component beta-binomial model are comparable fits to the data, this may imply there is not strong enough evidence of more than one group of chromosome arms. However, while the uni-component beta-binomial model and the two-component beta-binomial/binomial model appear to fit similarly, the two-component binomial model appears to be a slightly better fit than these two as shown by the corresponding posterior probabilities.

Maximum likelihood estimates obtained from fitting both the two-component beta-binomial and the beta-binomial/binomial model imply both components follow a binomial distribution as the dispersion parameter estimates are 0. Fits of all three two-component models yield identical parameter estimates, and therefore the rule obtained from the two-component binomial model which has the highest posterior probability is equivalent to that obtained from the other two-component models. Classification using this model places six chromosome arms in the TSG group. These are identified as chromosome arms 4q, 9p, 9q, 12q, 17p, and 18q. Note that three of these chromosome arms (4q, 9q and 12q) exhibit lower than the average background loss rate in the Barrett data set. However, 9p and 17p are categorized along with 5q in the TSG group. Furthermore, although not classified in the TSG group, chromosome arm 18q exhibits the fourth highest allelic-loss rate in the Barrett data set.

#### The Hammoud data set

**Figure 3:**
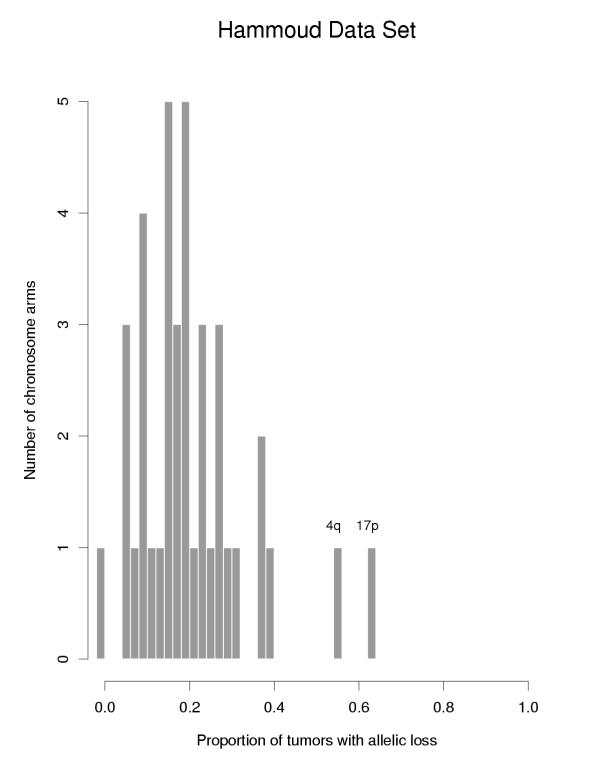
Histogram of allelic loss for the Hammoud data set

The Hammoud data set consists of 30 esophageal adenocarcinomas on 39 chromosome arms (the same arms included in the Gleeson data set). A histogram of the Hammoud data set is presented in Figure 3. Chromosome arms 4q and 17p have been identified on the plot as they appear to stand out from the others as having relatively high allelic-loss rates. The mean allelic-loss rate is 0.20 and the median allelic-loss rate is 0.18.

The pairwise comparisons using the Bayes factors for the Hammoud data set (See Table 4) demonstrate that both the two-component beta-binomial/binomial model and the two-component binomial model give the best fits to the data. Note that the posterior probabilities of these models are practically the same indicating these models are equally good fits to the data. As only two-component models are selected from the class, there is strong evidence to suggest that a second group of chromosome arms with TSGs exists. Classification using both the two-component beta-binomial/binomlal model and the two-component binomial model places chromosome arms 4q and 17p in the TSG group. Both models yield similar conditional probabilities of group membership for the arms, and as in the other data sets, both models yield the same classification rule. Note that chromosome arm 4q is implicated by our analysis of the Gleeson data set and 17p is implicated by our analyses of all three previous data sets.

## Conclusions

Testing of one versus two components in a mixture model is problematic as the likelihood ratio test is not applicable. Bayes factors provide a natural solution to this problem. Although we make only crude comparisons using the Bayes factors, the results favor the right model most of the time for data arising from a two-component model. More importantly, when comparing a two-component model versus a one-component model for these data, the two-component model is generally chosen.

For data that arise from a one-component beta-binomial model, the Bayes factors were not able to choose as well between the true model and a two-component model. Specifically, when comparing the true model to the two-component binomial, the false-positive rate was 16%. On the other hand, the Bayes factors are inconclusive for 62% of the data sets when making this comparison. This is actually encouraging when considering some frequentist options. Standard applicable frequentist methods such as an exact Monte Carlo test and the dispersion score test are limited to testing for one versus more than one group of chromosome arms [8,9]. Simulation studies examining these methods for these data reject the hypothesis of one group 93 and 89 percent of the time, respectively [10]. Based on this, one might conclude that a model with two (or more) groups would be appropriate. The results presented here would not support such a conclusion, at least most of the time. However, it is important to note that if such variability exists in the data as is expected and is ignored, the false-positive rate can be quite high. For example, if comparing a two-component binomial model and a one-component binomial model when there is only one group of chromosome arms exhibiting background loss, the two-component model would likely be favored. Thus, in practice it is recommended that several comparisons are made before selecting a model. In addition, it may be desirable to consider the posterior probabilities of all models jointly. When examining the posterior probabilities of each of the models for the four scenarios considered here, we found that the true model had the highest median posterior probability.

Table 3 summarizes the results of applying our approach to three esophageal adenocarcinoma data sets. It is important to note that a common locus on a chromosome arm was rarely chosen across the three studies. In fact, there were only a handful of loci that were investigated by at least two of the three data sets. Not surprisingly, chromosome arm 17p is chosen by the two-component models for all data sets as being in the TSG group. Chromosome arm 17p harbors a well known TSG called p53, which has been implicated in several cancers, including colon cancer, breast cancer and non-small cell lung cancer to name a few [1]. Also note that chromosome arm 9p is placed in the TSG group for the Barrett data set as well as the Gleeson data set. Similarly, chromosome arm 4q has been identified in both the Gleeson and Hammoud data sets. The Barrett data set also characterizes chromosome arm 5q as harboring a TSG, which has been previously identified in other studies as having a high frequency of allelic loss in colon cancer, non-small cell lung cancer, as well as renal cancer [1]. Similarly, 18q, identified in the Gleeson data set, is suspected of playing a causal role in colon cancer and osteosarcoma based on high allelic-loss frequencies there [1]. Also, chromosome arm 3p has been identified as having high loss in renal and non-small cell lung cancer [1]. The results from applying our methods to the three data sets differ somewhat from those of the previously published analyses. First a potential bias exists in the design of current allelic-loss studies, and is seen in the design of the Barrett and Gleeson studies. Chromosome arms suspected of harboring TSGs are evaluated at more loci than other arms. The proportion of tumors with allelic loss on an arm is then defined as the number of tumors with allelic loss at at least one of the informative loci divided by the number of tumors informative at at least one of the loci. For example in the Barrett study, one locus is investigated for most chromosome arms, but two loci are assessed for loss on arms 13q, 17p, and 18q. This increases the probability that allelic loss will be observed at those arms examined at two loci than at those examined at only one. To address this issue, our analysis considers only one locus (the most informative) per chromosome arm.

In the analysis presented by Barrett et al. (1996), the authors consider a uni-component binomial distribution for the background loss [1]. Frequencies falling far out in the tails of the binomial distribution, assuming a background loss rate of 0.23, correspond to chromosome arms with potential TSGs. However, it should be noted that the model upon which we base our results (two-component beta-binomial/binomial model) is selected over that assumed by Barrett et al. (1996), where our model has a corresponding posterior probability of 0.814 and the uni-component binomial has a posterior probability < 0.001 [1]. The results from Barrett et al. (1996) indicate that chromosome arms with significantly high loss rates are 5q, 9p, 13q, and 17p (with corresponding p-values < 0.05) [1]. Our approach also yields classification of 5q, 9p, and 17p in the TSG group. Although the fourth highest conditional probability corresponds to arm 13q, assuming a two-component beta-binomial/binomial model, the probability that it is in the TSG group is estimated to be quite low (0.084) with our approach. Barrett et al. (1996) also implicate chromosome arms 1p and 18q as potentially harboring TSGs (p-values < 0.10 and > 0.05) [1]. Our analysis demonstrates that these arms are not likely to be classified in the TSG group with conditional probabilities of 0.077 and 0.123, respectively.

The analytic approach employed by Gleeson et al. (1997) is to select a chromosome arm with a corresponding allelic-loss rate above an arbitrarily chosen cut-off of 50% as criterion for potentially harboring a TSG [11]. With this approach, Gleeson et al. (1997) implicate the following 10 chromosome arms; 3p, 4q, 5q, 8p, 9p, 9q, 12q, 13q, 17p, and 18q [11]. Our method gives the following conditional probabilities of harboring a TSG for these arms respectively: 0.003, 0.982, 0.327, 0.012, 0.916, 0.813, 0.859, 0.121, and 0.998. While our method also selects six of these arms, the conditional probability of the unselected four are estimated to be fairly low. Interestingly our conclusions regarding the Hammoud analysis correspond well to those of the authors. The criterion the authors used for selection of a chromosome arm into the TSG group was that the chromosome arm's allelic-loss rate should exceed two standard deviations above the observed mean allelic-loss rate. This approach is similar to that of Barrett et al. (1996) and more sound than that employed by Gleeson et al. (1997) as it assumes a reasonable model for the allelic-loss rate (in this case a normal distribution) and selects those outliers to the right of the distribution as suspicious [1,11]. Our approach, however, is more flexible in that multiple models consistent with the biological nature of the data are considered and compared and further, conditional probabilities of harboring a TSG are provided for each chromosome arm. For the arms selected by both us and Hammoud et al. (1996), the two arms selected, 4q and 17p, have conditional probabilities of 0.968 and 0.994 for harboring TSGs, respectively [4].

Results from the Bayes factors for the Gleeson data set are not completely clear. They cast doubt on whether the true underlying distribution really has two components or whether the two-component models chosen also provide a reasonable fit (relative to all the models considered) to overdispersed data exhibiting only background loss. Recall the simulation study where we demonstrate that for data arising from a uni-component beta-binomial model, the Bayes factors indicate that both the true model and the two-component binomial model are often both reasonable fits to the data. This motivates incorporating Bayesian model averaging (BMA) into the inference process [12]. An alternative would be to compute the posterior odds of a second component. First, the posterior probability of a two-component model could be obtained by averaging over the three two-component models. Second, the posterior probability of a uni-component model could be computed by averaging over the relevant uni-component models. The averaged Bayes factor would then be a ratio of the posterior probability of a two-component model to the posterior probability of a one-component model.

Furthermore, one could use Bayesian model averaging when estimating the conditional probability of group membership for each of the chromosome arms. Maximum likelihood estimates from different high probability models could lead to different inferences about parameters. Thus, this approach of averaging the conditional probability over the various models to classify the arms or weighting the parameter estimates by the posterior probability of a given model may be more desirable than choosing a single best model from which to make inference. Specifically, one could weight estimates by *P*(*H*_*j*_|***X***). For example, suppose chromosome arm 13q is suspected of harboring a TSG from past experiments and we desire a probability that *Z*_13*q *_= 1 based on these data. Because of model uncertainty we may be hesitant to compute the probability based solely on one model. Instead, we could estimate this probability as:


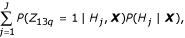


where *j *indexes over all of the models considered. This is a potential alternative to classifying the chromosome arms using the classical maximum likelihood approach that needs to be further explored. It is interesting to note that the two-component beta-binomial mixture model was never chosen for any of the data sets. Although it was certainly favored over the one-component binomial model in all data sets and over the uni-component beta-binomial model in the Barrett data set, it was never chosen to be in the set of candidate models. The class of models considered here is based on our beliefs of the biology of the data. However, the ability to screen the tumor cell genome for chromosome arms which harbor TSGs lies in a better understanding of the background distribution. Characterizing the background distribution would allow a more definitive identification of arms exhibiting abnormal loss.

## Methods

### Data

The three data sets to which we apply our methods were previously published and analyzed using other techniques [1,4,11].

### Computing Bayes factors for the proposed class of mixture models

Computing Bayes factors can be challenging as non-trivial integration is often required to estimate the marginal probabilities under each model considered. Specifically, calculating Bayes factors involves integrating the likelihood over the entire parameter space for each model considered. Thus, the integrals tend to be high-dimensional. In general, we need to compute

*I *= ∫ *Pr*(***X***|***λ***, *H*)*π*(***λ***|*H*)*d****λ***.

This can be quite computationally intensive. When the integral is of high dimension (> 6), quadrature methods can be unreliable [13]. In addition, and more relevant to our situation, for moderate to large sample sizes (> 35), numerical methods can be both inefficient and unreliable [7,14]. An alternative approach is to use Gibbs sampling techniques. However, for mixture models, these methods often miss important mass as the chain tends to get stuck near one mode resulting in an underestimate of the integral [14]. Furthermore, because the sampling is not independent, there is no simple way of self-monitoring convergence.

Another method of estimating integrals is simple Monte Carlo, that involves sampling from the prior distribution, *π*(***λ***). The simple Monte Carlo estimate of the integral is the averaged likelihood at the sampled parameter values or


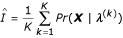


This has been shown to be a good estimate for likelihoods that are relatively flat. However, if the posterior is concentrated relative to the prior, the variance of the estimate will be large, and convergence to a Gaussian will be slow [7]. Thus, sampling from the prior distribution is often not very efficient. A potential solution to this problem is to do importance sampling that involves sampling from π*(***λ***), the importance sampling function [7,14]. The estimate then becomes


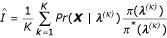


where 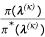 is known as the importance sampling ratio. The simple Monte Carlo estimate is a special case of importance sampling where π*(·) is chosen to be the prior distribution. However, the importance sampling estimate can be an improvement over the simple Monte Carlo estimate if π*(·) is chosen such that the sampling is more efficient, e.g., if π*(·) is centered around the mass. There has been some success with importance sampling in a non-mixture model setting [14].

Our solution is to first write the likelihood in its complete-data form. The likelihood for the mixture of two beta-binomial distributions is written as follows:


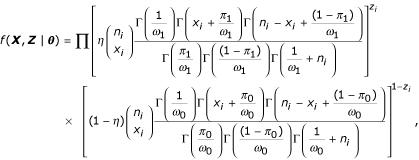


where ***z ***= (*z*_1_, *z*_2_,...,*z*_*N*_)^*T *^and the *z*_*i*_s are unobserved group membership indicators such that *z*_*i *_= 0 if *x*_*i *_is from the background component and *z*_*i *_= 1 if *x*_*i *_is from the TSG component. Then the marginal probability of ***X ***becomes


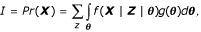


where *I *denotes the marginal probability of the data (or integrated likelihood) and where *g *is the prior distribution of ***θ***.

We then estimate this integral using a method we developed called the Uniform Distance Method (UDM). This method is a variant on importance sampling and involves a combination of either quadrature or exact integration and sampling of the membership vectors, ***Z***. The idea behind the method is to use *P*(***Z***|***θ ***= 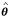, ***x***) where 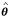 is the MLE of ***θ ***to provide information on the important groupings, i.e., which chromosome arms are likely to be clustered together. While the membership vectors are sampled independently, the membership values within a group are sampled dependently, making these groupings more likely to be maintained than if the values were sampled independently.

The development and assessment of UDM is discussed in detail in Desai (2000) and demonstrates solid performance in estimating these integrals [10]. Software for implementing the method is available by contacting the first author. Note that for all analyses presented in this paper, uniform priors are assumed for the unknown parameters.

## Abbreviations

TSG, tumor suppressor gene; MLE, maximum likelihood estimate; 2 bb, two-component beta-binomial model; 2 bb/bin, two-component beta-binomial/binomial model; 2 bin, two-component binomial model; 1 bb, uni-coniponent beta-binomial model; 1 bin, uni-component binomial model; BMA, Bayesian model averaging; UDM, uniform distance method

## Authors contributions

Both MD and MJE contributed substantially to the development of the models and the methodology. MD performed the simulation study and analysis of the three data sets. Both authors have read and approved the final version of the manuscript.

## Figures and Tables

**Table 1:** Description of scenarios used in simulation study

		Loss Rates	
Scenario	Model*	Non-TSG** group	TSG group
1	Two-component binomial mixture	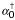 = 0.22 (33 arms)	*α*_1 _= 0.66 (5 arms)
2	Uni-component beta-binomial	*α*_0 _~ *β*(0.26, 0.07) (38 arms)	-
3	Two-component multi-binomial/binomial mixture	*α*_0 _= 0.22 (33 arms)	*α*_1 _= (1, 0.80, 0.64, 0.43, 0.43) (5 arms)
4	Two-component multi-binomial/beta-binomial	*α*_0 _~ *β*(0.26, 0.07) (33 arms)	*α*_1 _= (1, 0.80, 0.64, 0.43, 0.43) (5 arms)

* Model from which data were generated

** TSG: Tumor suppressor gene

^† ^*α*_0_: loss rate for non-TSG group. *α*_1_: loss rate for TSG group

**Table 2:** Percentage of time model under *H*_1 _is favored over model under *H*_0 _for different scenarios For a given scenario, the rows indicate the model under *H*_1 _while the columns indicate the model under *H*_0_. The (*i*, *j*)th element in the matrix represents the percentage of time the model in the *i*th row is favored over that in the *j*th column.

Scenario 1 (*α*_0 _= 0.22, *α*_1 _= 0.66)					
*H*_1_/*H*_0_	2 bin*	2 bb/bin	2 bb	1 bb	1 bin
2 bin	0	21	75	81	100
2 bb/bin	10	0	80	80	100
2 bb	5	0	0	50	98
1 bb	5	0	0	0	100
1 bin	0	0	0	0	0

Scenario 2 (*α *_0 _~ *β *(0.26,0.07))					

*H*_1_/*H*_0_	1 bb	2 bin	2 bb/bin	2 bb	1 bin
1 bb	0	22	21	49	75
2 bin	16	0	24	44	72
2 bb/bin	7	14	0	26	74
2 bb	0	12	0	0	68
1 bin	7	0	7	18	0

Scenario 3 (*α*_0 _= 0.22, *α*_1 _= (1, 0.80, 0.64, 0.43, 0.43))					

*H*_1_/*H*_0_	2 bb/bin	2 bb	2 bin	1 bb	1 bin
2 bb/bin	0	78	79	98	100
2 bb	1	0	31	100	100
2 bin	0	28	0	87	100
1 bb	0	0	5	0	100
1 bin	0	0	0	0	0

Scenario 4 (*α*_0 _~ *β *(0.26, 0.07), *α*_1 _= (1, 0.80, 0.64, 0.43, 0.43))					

*H*_1_/*H*_0_	2 bb	2 bb/bin	1 bb	2 bin	1 bin
2 bb	0	35	75	97	100
2 bb/bin	9	0	54	99	100
1 bb	0	5	0	72	100
2 bin	0	0	9	0	100
1 bin	0	0	0	0	0

*2 bb: Two-component beta-binomial.

2 bb/bin: Two-component beta-binomial/binomial.

2 bin: Two-component binomial.

1 bb: One-component beta-binomial.

1 bin: One-component binomial.

**Table 3:** Summary of results after applying methods to three data sets For each data set, the selected model(s) with the chromosome arms classified in the tumor suppressor gene group and corresponding conditional probabilities of harboring a tumor suppressor gene are provided. A set of models was chosen such that models in the set had 2*ln*(Bayes factors) exceeding 2 when compared to models outside the set and 2*ln*(Bayes factors) less than 2 when compared to models within the set. A chromosome arm is in bold print if it has been identified in more than one data set.

Data Set	Model Chosen	Chromosome Arms Classified in TSG* Group (conditional probability)
Barrett	2 bb/bin	5q (1), **9p**(0.962), **17p**(1)
Gleeson	2 bb/bin	**4q**(0.982), **9p**(0.916), 9q (0.813), 12q (0.859), **17p **(0.998), 18q (0.954)
	2 bin	**4q**(0.982), **9p**(0.916), 9q (0.813), 12q (0.859), **17p **(0.998), 18q (0.954)
	1 bb	none
Hammoud	2 bb/bin	**4q **(0.968), **17p **(0.994)
	2 bin	**4q **(0.989), **17p **(0.998)

* TSG: tumor suppressor gene

2 bb: Two-component beta-binomial

2 bb/bin: Two-component beta-binomial/binomial

2 bin: Two-component binomial

1 bb: One-component beta-binomial

1 bin: One-component binomial

**Table 4:** 2*ln*(Bayes Factors) and posterior probabilities of each model considered for the three data sets For a given data set, the first five rows of data correspond to the model under *H*_1 _while the first five columns correspond to the model under *H*_0_. The (*i*, *j*)th element in the matrix represents the value of 2*ln*(Bayes Factors) for the model corresponding to the *i*th row versus the model corresponding to the *j*th column. Values of 2*ln*(Bayes Factors) are in bold print if they exceed 2. The last column provides values of the posterior probability of the model in the *i*th row. Those values corresponding to selected models are in bold print.

Barrett data set						
*H*_1_/*H*_0_	2 bb*	2 bb/bin	2 bin	1 bb	1 bin	Post.Prob**

2 bb	0	-4.398	-0.114	**12.144**	**45.281**	0.090
2 bb/bin	**4.398**	0	**4.284**	**16.542**	**49.679**	**0.814**
2 bin	0.114	-4.284	0	**12.258**	**45.395**	0.096
1 bb	-12.144	-16.542	-12.258	0	**33.137**	< 0.001
1 bin	-45.281	-49.679	-45.395	-33.137	0	< 0.001

Gleeson data set						

*H*_1_/*H*_0_	2 bb	2 bb/bin	2 bin	1 bb	1 bin	Post.Prob.
2 bb	0	-2.173	-3.390	-2.065	**6.705**	0.066
2 bb/bin	**2.173**	0	-1.724	0.108	**8.878**	**0.194**
2 bin	**3.390**	1.724	0	1.832	**10.601**	**0.460**
1 bb	**2.065**	-0.108	-1.832	0	**8.770**	**0.276**
1 bin	-6.705	-8.878	-10.601	-8.770	0	0.003

Hammoud data set						

*H*_1_/*H*_0_	2 bb	2 bb/bin	2 bin	1 bb	1 bin	Post.Prob.
2 bb	0	-3.514	-3.513	-1.114	**5.951**	0.070
2 bb/bin	**3.514**	0	0.020	**2.400**	**9.465**	**0.404**
2 bin	**3.513**	-0.020	0	**2.380**	**9.444**	**0.400**
1 bb	1.114	-2.400	-2.380	0	**7.064**	0.122
1 bin	-5.951	-9.465	-7.064	-7.064	0	0.004

*2 bb: Two-component beta-binomial

2 bb/bin: Two-component beta-binomial/binomial

2 bin: Two-component binomial

1 bb: One-component beta-binomial

1 bin: One-component binomial

** Post.Prob.:Posterior probability

**Table 5:** Results from fitting two-component models to the Barrett data set Maximum likelihood estimates along with selected chromosome arms and corresponding conditional probabilities of harboring a tumor suppressor gene for the two-component models for the Barrett data set.

Model	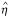	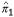	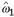	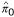	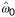	Arms classified in TSG^† ^group (conditional probability)
2 bb/bin*	0.097	0.708	0.487	0.228	-	5q (1); 9p(0.962); 17p(1)
2 bin	0.073	0.827	-	0.230	-	5q (1); 9p(0.93); 17p(1)
2 bb	0.097	0.708	0.487	0.228	0.000	5q (1); 9p(0.962); 17p(1)

^†^TSG: tumor suppressor gene

*2 bb; Two-component beta-binomial

2 bb/bin: Two-component beta-binomial/binomial

2 bin: Two-component binomial
